# Evaluation of *Annona muricata* extract against *Staphylococcus aureus* isolate and in-silico activity of bioactive compounds against Capsular protein (Cap5O)

**DOI:** 10.1186/s12906-022-03672-4

**Published:** 2022-07-19

**Authors:** Uwem Okon Edet, Francisca Obiageri Nwaokorie, Elizabeth Nkagafel Mbim, Edet Effiong Asanga, Yeneochia Ogar Agbor, Henshaw Uchechi Okoroiwu, Bassey Okon Edet, Nikita Umoafia, Ani Nkang

**Affiliations:** 1 Department of Biological Science (Microbiology Unit), Faculty of Natural and Applied Sciences, Arthur Jarvis University, Akpabuyo, Cross River State Nigeria; 2 Department of Medical Laboratory Science, College of Medicine, University of Lagos, Lagos, Lagos State Nigeria; 3 Department of Public Health, Faculty of Basic Medical Sciences, Arthur Jarvis University, Akpabuyo, Cross River State Nigeria; 4 Department of Chemical Sciences (Biochemistry Unit), Faculty of Natural and Applied Sciences, Arthur Jarvis University, Akpabuyo, Cross River State Nigeria; 5 Department of Medical Laboratory Sciences, Faculty of Basic Medical Sciences, Arthur Jarvis University, Akpabuyo, Cross River State Nigeria

**Keywords:** Docking, Nutrients, Phytochemicals, *Annona muricata*, MDRSA, Cap5O

## Abstract

**Background:**

*Staphylococcus aureus* has prevailed against the majority of antibiotics currently in clinical use, making it a significant global public health problem. As a safer alternative, bioactive compounds have been explored. *Annona muricata* has been shown to possess antimicrobial activity. However, there are few reports on the molecular activity of *A. muricata* bioactive compounds against *S. aureus*. Thus, this study was aimed at evaluating the antimicrobial activity of its crude extract as well as investigating the potential of its bioactive compounds against the Cap5O capsular polysaccharides (CPS) of *S. aureus* via molecular docking.

**Methods:**

Collection of plant leaves, preparation of extracts, anti-nutrient analysis, phytochemical screening via crude method and gas chromatography-mass spectrophotometer (GC-MS), isolation and characterization of *S. aureus* and the antimicrobial activity test were all done using standard protocols. Molecular docking was done using the MCULE online tool with emphasis on docking scores, toxicity, and other properties.

**Results:**

Crude screening of the extracts showed the presence of polyphenols, hydroxyanthraquinones, reducing compounds, flavonoids, saponins, glycosides, alkaloids, anthraquinones, phlobatannins and tannins in different concentrations. Anti-nutrient analysis showed the presence of allowable levels of evaluated anti-nutrients. GC-MS revealed a total of twenty-nine (29) bioactive compounds, out of which only 4 (13.80%) docked without toxicity and these were bicyclo[4.1.0]heptan-2-one 6-methyl, trichloromethane, carbonic acid 2-dimethylaminoethyl propyl ester, and 1-methyl-4-phenyl-5-thioxo-1,2,4-triazolidin-3-one on either the NAD-binding or C-terminal substrate binding domain of Cap5O.

**Conclusion:**

Results obtained show that Cap5O could be a potential drug target for multi-drug resistant *S. aureus*, however, further studies aimed at evaluating these bioactive compounds individually and in combination are highly needed.

**Supplementary Information:**

The online version contains supplementary material available at 10.1186/s12906-022-03672-4.

## Background

*S. aureus* is a well-characterized human pathogen with an outstanding ability to outwit our immune system [1]. Its multi-drug resistance ability makes it one of the most intractable pathogens and a significant public health challenge of our time. Since the isolation of the first multi-drug resistant *S. aureus* (MRSA) in the 1960s [2], it has been reported in Africa [3, 4], Europe [5], Asia [6], and America [7]. It has practically prevailed against all antibiotics introduced into clinical practice since the 1940s [1, 2]. Given the global importance of antibiotics resistance, the World Health Organization (WHO) published its first-ever list of antibiotics resistant priority pathogens for which new antibiotics are urgently needed, and this included *S. aureus* alongside *Enterococcus faecium*, *Helicobacter pylori*, *Campylobacter* spp., *Salmonella*, and *Neisseria gonorrhoeae* [8]. There is an urgent need for new antibacterial agents to combat the global public health threat posed by *S. aureus* and other pathogens [9–11]. As a result of the urgency, several alternatives including the use of phytochemicals have been proposed. These metabolites include but are not limited to members of alkaloids, flavonoids, quinones, polyphenols, saponins, and tannins families [12–17].

The genus *Annona* has over 70 species amongst which *A. muricata* is the most widely grown in West, Central and Eastern parts of Africa, Southeast Asia, and Central and South America [17]. In addition to the edible fruit pulp, numerous uses have been found for the plant and these include the treatment and management of pains, fever, infections (bacterial and parasitic), skin diseases, hypertension, inflammation, diabetes, and cancer [7, 17]. The antimicrobial activity of the leaf and bark extracts of the *A. muricata* has been tested against MRSA using methods that cannot identify potential drug targets [7].

As part of the rational drug design, molecular docking has emerged as an in-silico approach to drug discovery. It allows for the discovery and identification of lead compounds with therapeutic potentials by allowing efficacy evaluation, molecular interaction prediction, toxicity or adverse effect prediction, drug repurposing, target fishing and profiling, and it is adaptable to artificial intelligence [18, 19]. One of the virulence factors of *S. aureus* is its CPS Cap5O, an important protein in the biosynthesis of serotype 5 capsular polysaccharides, which interfere with the crucial interaction between the bacteria and eukaryotic cells (phagocytic and non-phagocytic) making them evade immune system via mechanisms that are not well known [20]. In other studies, it is proposed to enhance the biosynthesis of the peptidoglycan of the cell wall as well as aid *S. aureus* in the evasion of the host immune system [21, 22]. However, there are few reports on the molecular activity of *A. muricata* bioactive compounds against *S. aureus,* none evaluated their drug potential. Therefore, this study was aimed at evaluating the antimicrobial activity of the crude extract in addition to the molecular docking potentials or druggability (score, toxicity, and Lipinski’s rule of 5 amongst others) of its various bioactive compounds against Cap5O capsular protein.

## Methods

### Collection of plant leaves

Fresh leaves of *A. muricata* were randomly collected from three different mature plants growing in and around Nyakasang Village, Atimbo, Cross River State, Nigeria. The leaf was identified by a Botanist Professor Ani Nkang and identity number AJU/2021/12 assigned. The leaves were processed as previously reported [9, 10].

### Preparation of the extract

For this study, two types of extracts were prepared using methods previously reported and these were aqueous (distilled water) and ethanolic (analytical grade) [9, 10]. To 200 ml of the respective solvents, 20 g of the pulverized powder were added to obtain a ratio of 1:10. The mixtures were then placed on an electrical orbital shaker operating at a speed of 200 rpm for 24 h at a temperature of 28 ± 2 °C to enhance the extraction efficiency. After overnight soaking in the solvents, filtration was performed using a clean Whatman filter paper (No.1). The resulting residues were subjected to the same process thrice to ensure all the phytochemicals go into solution. The final filtrates were then concentrated to slurries using an electric water bath operated at 50 °C to obtain yields of 1.15 g, (5.75%) and 1.05 g (5.25%) of aqueous and ethanol extract, respectively. These were then stored using sterile and moisture-free Bijou bottles at room temperature (28 ± 2 °C) until needed for further analysis.

### Qualitative phytochemical and anti-nutrients evaluations

A total of ten (10) phytochemicals were evaluated as previously reported. They were alkaloids [23], glycosides and saponins [24], tannins and anthraquinones [25], reducing compounds and polyphenol [26], flavonoids, and phlobatannins [27], and hydroxymethyl anthraquinones [28]. Anti-nutrients were also evaluated as previously reported [29].

### Gas chromatography-mass spectrometry analysis

This was performed as described previously [9]. Similar operational conditions were utilized in the study. The relative percentage amount of each component was calculated by comparing its average peak area to the total area. TurboMass Ver 5.2.0 software was employed to handle mass spectra and chromatograms. The interpretation was done using the NIST14 library.

### Collection and processing of skin swabs

Following informed consent, skin swabs were collected from students (*n* = 5) aged 17–22 years without visible signs of any skin disease and not on any antibiotic medication using a sterile disposable skin swab stick for each participant. The skin swabs were collected early in the morning and just before bedtime bath to allow for an adequate proliferation of the test organism.

### Culturing of the test microorganisms

The samples were inoculated onto freshly prepared mannitol salt agar (MSA) using surface streaking and incubated at 37 °C for 18–24 h after which microbial load was evaluated. Following the growth of the test organism (golden yellow colonies), discrete colonies were sub-cultured onto plates containing freshly prepared sterile nutrient agar and incubated at 37 °C for 24 h. Purified isolates were also maintained on nutrient agar slants for further purposes.

### Antibacterial activity of *A. muricata*

The antimicrobial sensitivity was carried out using both the disk diffusion method (Cefoxitin) as well as the tube method for the extract. Both were done as previously described by Clinical Laboratory Standard Institute (CLSI) [30]. In addition, the tube dilution method was also used to evaluate the sensitivity of the isolates via turbidity measurements at 540 nm. Briefly, approximately 1.0 g of *A. muricata* slurry was used to perform a ten-fold dilution, and to dilutions 10^− 1^, 10^− 2^, 10^− 3^ and 10^− 4^, 1 ml each of the extract was taken and introduced into test tubes containing 4 ml of an overnight culture of test isolate, and absorbance measured at 540 nm after 24 h. For the disk diffusion method, test organisms were inoculated into tubes containing sterile peptone water and incubated for 18 h at 37 °C. Following incubation, the inoculum was adjusted to 0.5 McFarland standard (approximately 1.5 × 10^8^ CFU/ml) and vortexed to ensure proper mixing. Consequently, fresh sterile cotton-tipped swabs were dipped into the suspension, and excess liquid from the swabs removed by pressing it against the sides of the tubes. The swabs were then inoculated onto freshly prepared MSA with holes bored on the plates to which 1 ml of different concentrations of extract (10^− 1^, 10^− 2^, 10^− 3^, and 10^− 4^) respectively were introduced aseptically and allowed to stand for 24 h. This was repeated for all test isolates and a conventional antibiotic (cefoxitin), a control was also set up. All determinations were done in triplicates.

### Molecular docking of ligands and protein Cap5O

Molecular docking was performed using the 1-click docking tool hosted online at MCULE.com. A total of twenty-nine (29) compounds obtained from GC-MS were utilized as ligands. The Cap5O structure was retrieved from the RCSB protein database. The ligands were first prepared by converting them into pbd format. Next, the structure of the Cap5O was retrieved from the MCULE website and confirmed using the Protein Data Bank (PDB code: PDB ID: 3ojl) online tool. The docking was done blindly, that is, without any prior knowledge of the catalytic or binding sites of Cap5O. The ligands were then docked onto the Cap5O protein for docking scores and poses. After which the toxicity profiles and pharmacological parameters of the ligands were also obtained.

### Ethical statement

Ethical clearance for the study was sought for and obtained from the research and ethical committee of Arthur Jarvis University with reference number REC05/07/22 J03. To the participants, the ethical statement was made available to them and their informed consent was also obtained verbally and in writing before the commencement of the study. Confidentiality was maintained by not labeling the samples with participant names. All methods were carried out in accordance with relevant guidelines and regulations (declaration of Helsinki).

### Statistical analysis

All the data generated from the microbiological analysis and phytochemical quantification were managed using Microsoft Excel Office 2016 and analyzed using simple descriptive statistics (mean, percentages, and standard deviation determinations as applicable).

## Results

### Phytochemical evaluation of *Annona muricata*

The result of the qualitative phytochemical evaluation of the ethanolic (E.L.E) and aqueous (A.L.E) extracts of *A. muricata* revealed the presence of polyphenols, hydroxyanthraquinones, reducing compounds, flavonoids, saponins, glycosides, alkaloids, anthraquinones, phlobatannins and tannins (Table 1). Polyphenols was the most abundant phytochemical in both extracts.

**Table 1:** Phytochemical screening of the bark and leaves extracts of *A. muricata*

Phytochemicals	E.L E	A.L E
Alkaloids	**++**	**+**
Glycosides	**++**	**+**
Saponins	**+**	**++**
Tannins	**+**	**_**
Flavonoids	**++**	**+**
Reducing compounds	**++**	**+**
Polyphenols	**++**	**+++**
Phlobatannins	**_**	**+**
Anthraquinones	**+**	**+**
Hydroxyanthraquinone	**+**	**++**

Key: *E.L.E* ethanol leaves extract, *A.L.E* aqueous leave extract

### Levels of anti-nutrients

The result of the anti-nutrients is shown in Table 2. Total oxalate had the highest concentration of 268.08 mg/100 g of dry matter followed by soluble oxalate with a concentration of 178.55 mg/100 g of dry matter. The least abundant anti-nutrient was phytate with a concentration of 5.45 mg/100 g per dry matter.

**Table 2:** Level of anti-nutrients in leaves of *A. muricata* (mg/100 g)

No. of analysis	Phytate	Total oxalate	Soluble oxalate
1	5.45	268.07	176.55
2	5.46	268.10	176.54
3	5.44	268.08	176.55
Mean ± SD	5.45 ± 0.01	268.08 ± 002	176.55 ± 0.01

### GC-MS analysis of *Annona muricata* leaves

The result of the GC-MS revealed the presence of 29 bioactive compounds as shown in Table 3. In addition to the nomenclatures of the compounds, their retention times, and areas (concentrations).

**Table 3:** Bioactive compounds (ligands), docking scores, and toxicity

SN	Compounds (Ligands)	RT	Area	Docking scores (best score)	Toxicity
1	Trichloromethane	6.37	0.91	− 2.4-2.7 (2.7)	None
2	Bicyclo[4.1.0]heptan-2-one 6-methyl-	29.58	1.00	−4.3-4.8(4.8)	None
3	1-Tetradecyne	25.43	0.48	−4.6-4.9	Yes
4	Citronellylisobutyrate	30.12	0.48	−4.5-4.8	Yes
5	Hexadecanoic acid, methyl ester	30.62	17.31	−4.3-4.4	Yes
6	9-Octadecenoic acid, methyl ester, (E)-	32.13	50.88	−4.0-4.2	Yes
7	Methyl stearate	32.31	16.65	−4.3-4.4	Yes
8	1-methyl-4-phenyl-5-thioxo-1,2,4-triazolidin-3-one	32.47	0.58	−4.9-5.5 (5.5)	None
9	Carbonic acid, monoamide, N-(2-ethylphenyl)-, propyl ester	32.76	0.34	−3.2-3.5 (3.5)	None
10	Heptasiloxane, 1,1,3,3,5,5,7,7,9,9,11,11,13,13-tetradecamethyl-	33.73	2.2411	Na	None
11	2-(Acetoxymethyl)-3-(methoxycarbonyl)biphenylene	33.44	0.87	−6.3-6.5	Yes
12	Silicic acid, diethyl bis(trimethylsilyl) ester	33.48	1.01	Na	Yes
13	Silicic acid, diethyl bis(trimethylsilyl) ester	33.48	1.01	Na	Yes
14	Carbonic acid, monoamide, N-methyl-N-phenyl-, 2-methylpropyl ester	33.54	0.49	5.1	Yes
15	Phenanthridinium, 5,6-dimethyl-, iodide	33.64	1.09	Na	Yes
16	Pyridine-3-carboxylic acid, 1,4-dihydro-5-cyano-2-hydroxy-4-(4-isopropylphenyl)-6-methyl-, ethyl ester	33.66	0.84	−6.7-7.3	Yes
17	2-(Acetoxymethyl)-3-(methoxycarbonyl)biphenylene	33.44	0.87	6.4–6.8	Yes
18	Octasiloxane, 1,1,3,3,5,5,7,7,9,9,11,11,13,13,15,15-hexadecamethyl-	34.19	0.97	6.4–6.8	Yes
19	Silicic acid, diethyl bis(trimethylsilyl) ester	34.97	1.06	Na	Yes
20	Heptasiloxane, 1,1,3,3,5,5,7,7,9,9,11,11,13,13-tetradecamethyl-	34.98	0.77	Na	Yes
21	1,2-Bis(trimethylsilyl)benzene	37.25	0.78	Na	Yes
22	Octasiloxane, 1,1,3,3,5,5,7,7,9,9,11,11,13,13,15,15-hexadecamethyl-	33.27	1.19	Na	Yes
23	Octasiloxane, 1,1,3,3,5,5,7,7,9,9,11,11,13,13,15,15-hexadecamethyl-	33.27	1.19	Na	Yes
24	Octasiloxane, 1,1,3,3,5,5,7,7,9,9,11,11,13,13,15,15-hexadecamethyl-	33.27	1.19	Na	Yes
25	Indole-2-one, 2,3-dihydro-N-hydroxy-4-methoxy-3,3-dimethyl-	33.48	1.01	5.6–6.3	Yes
26	1,2-Bis(trimethylsilyl)benzene	35.07	1.18	Na	Yes
27	2-(Acetoxymethyl)-3-(methoxycarbonyl)biphenylene	36.68	0.11	−6.3-6.8	Yes
28	Tricyclo[4.2.1.0(2,5)]non-7-ene, 3,4-di(tris(trimethylsilyloxy)silyl)-	36.85	0.24	Na	Yes
29	Silicic acid, diethyl bis(trimethylsilyl) ester	36.90	0.12	Na	Yes

For some of the bioactive compounds, since they were toxic, no docking scores were generated for them apart from heptasiloxane, 1,1,3,3,5,5,7,7,9,9,11,11,13,13-tetradecamethyl- that did not return any docking output

Key: *Na* Not applicable

### In vitro antibacterial activity of *A. muricata* leaves

Table 4 shows the results of the antimicrobial activity done via the disk and well diffusion using the extract and cefoxitin. The mean zones of inhibition ranged from 12.20 to 15.56 mm for the strongest (10^− 1^) dilution while for 10^− 2^, the range was 6.45 to 9.25 mm, and for 10^− 3^ and 10^− 4^ dilution, the isolates showed no sensitivity. Table 5 shows the results of the tube assay for the antibacterial activity of the *A. muricata* and that of cefoxitin as measured by absorbance at 540 nm. The cefoxitin stock solution showed turbidity that was slightly lower than that of the highest extract concentration with values that ranged from 0.12 to 0.18 and 0.23 to 0.48 mm, respectively. Compared to other extract concentrations, the absorbance readings were higher implying that these concentrations were less bactericidal in action.

**Table 4:** Zones of inhibition of *A.muricata*extracts and cefoxitin (mm)

Samples	10^−1^	10^− 2^	10^−3^	10^−4^	Cefoxitin
1	14.25	9.25	NI	NI	21.50
2	15.56	7.40	NI	NI	19.25
3	13.25	6.50	NI	NI	24.50
4	12.20	6.45	NI	NI	23.50
5	14.70	7.60	NI	NI	17.30

Key: *NI* No inhibition

**Table 5:** Antibacterial activity of *A.muricata* leaves and cefoxitin in peptone water at OD_540_nm

Skin swab samples	Various conc. of the extracts				
	Stock	10^**−1**^	10^**− 2**^	10^**−3**^	10^**−4**^
1	2.12 ± 0.01	0.34 ± 0.01	0.98 ± 0.03	1.51 ± 0.02	1.67 ± 0.01
2	1.71 ± 0.02	0.24 ± 0.01	1.25 ± 0.01	1.61 ± 0.03	1.84 ± 0.02
3	2.58 ± 0.03	0.23 ± 0.02	1.19 ± 0.02	1.59 ± 0.01	1.79 ± 0.03
4	1.73 ± 0.01	0.28 ± 0.01	1.03 ± 0.03	1.62 ± 0.01	1.73 ± 0.01
5	2.13 ± 0.02	0.48 ± 0.01	1.12 ± 0.01	1.45 ± 0.03	1.69 ± 0.02

### Molecular docking analysis

**Fig. 1:**
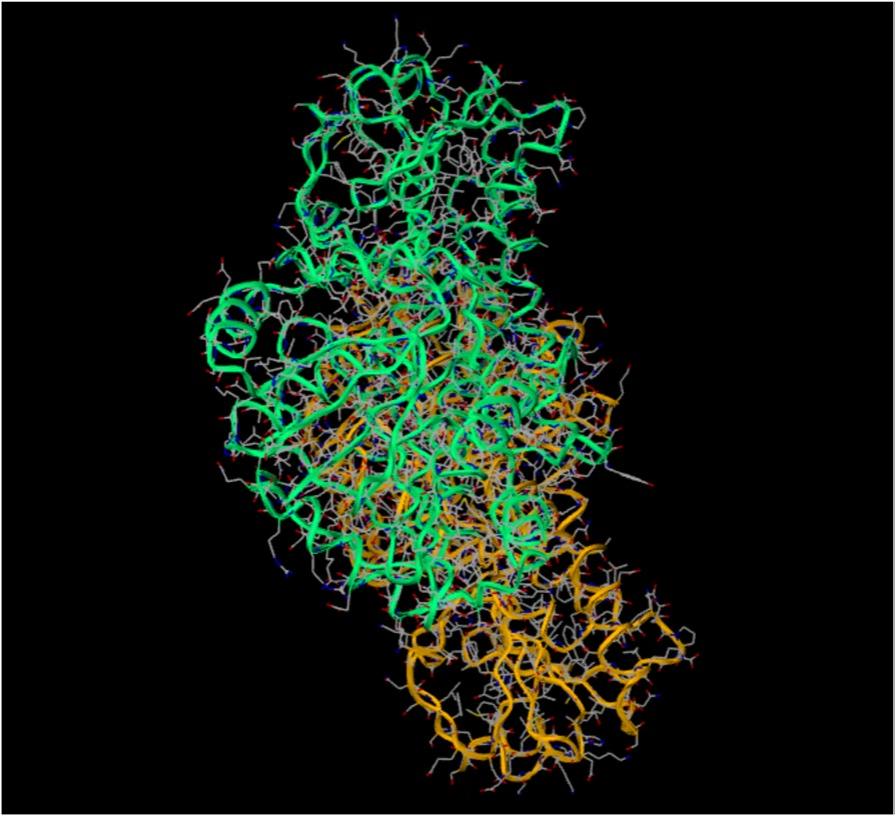
3D structure of Cap5O

**Fig. 2:**
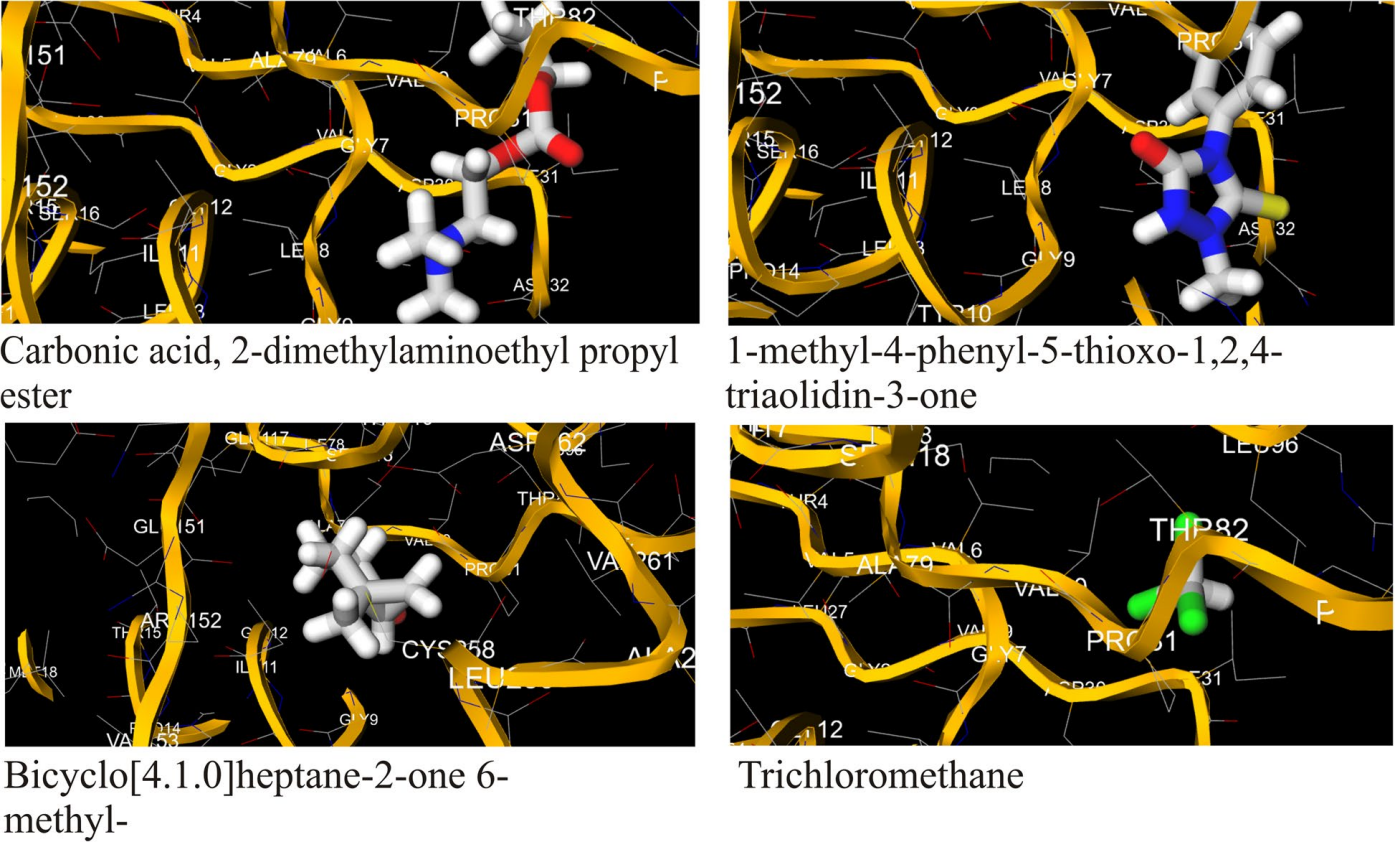
Docking pose of selected ligands with no toxicity against Cap5O showing residues

The bioactive compounds obtained via GC-MS were utilized as ligands for molecular docking with the protein, Cap5O to determine the binding interactions or poses and toxicity using the MCULE tool. Out of the 29 bioactive compounds, only 4 (13.79%) showed non-toxic interactions with the study protein (Table 3). Compounds that returned non-toxic poses were carbonic acid, 2-dimethylaminoethyl propyl ester, 1-methyl-4-phenyl-5-thioxo-1,2,4-triazolidin-3-one, bicyclo[4.1.0]heptan-2-one, 6-methyl- and trichloromethane. Figure 1 shows the three-dimensional structure of the CPS Cap5O protein of *S. aureus* while Fig. 2 shows the interactions of the ligands with the Cap5O protein together with the amino acid (AA) residues*.* As can be seen from the complexes formed between the protein and the bioactive compounds in Fig. 2, the bindings occurred at various points and with different AA residues. For trichloromethane, the interaction occurred with two AA residues namely threonine (THR 82) and proline (PRO 31). Similarly, bicyclo[4.1.0]heptan-2-one 6-methyl, also interacted with two amino acid residues which were glycine (GLY 9) and tyrosine (TYR 10). With bioactive compound 1-methyl-4-phenyl-5-thioxo-1,2,4-triazolidin-3-one, the amino acids residues were isoleucine (ILL 11), glycine (GLY 11), alanine (ALA 70), glycine (GLY 12) and proline (PRO 150). Carbonic acid monoamide N-(2-ethylphenyl) propyl ester interacted with amino acid residues glycine (GLY12), Isoleucine (ILL11), alanine (ALA 79), cysteine (258) and leucine (LEU 259). Extended details of docking results using the same tool are presented as supplementary tables (Tables S1 and S2). These tables show the molecular weights of the non-toxic bioactive compounds, the number of H-bond donors, H-bond acceptors, and the calculated Log *P* values amongst other parameters.

## Discussion

In addition to its edible fruits, *A. muricata* leaves are widely exploited for their medicinal properties. The high levels of evaluated anti-nutrients in our study were comparatively higher than those of commonly edible leafy vegetables in Nigeria, and this could explain why its leaf is not popular as edible vegetables such as *Dennettia tripetala* and *Lasianthera africana* in Nigeria [10]. Some of its documented medicinal properties include the management of various microbial infections such as diarrhea, pneumonia, skin infections, and urinary tract infections among others [31]. Our study showed the presence of various amounts of bioactive compounds belonging to the families of phytochemicals namely phenols, tannins, saponins, flavonoids, and polyphenols among others in both extracts of the plant. The presence of these compounds has been linked to the reported antimicrobial activity of our studied medicinal plant [10, 31, 32]. Polyphenols have been shown to exert their inhibitory properties on pathogenic microorganisms via inhibition of their virulence factors [32]. In other studies, these phytochemicals have been reported to act synergistically with some antibiotics against a few pathogens [33, 34]. Similarly, it has been shown that titanic acid which constitutes a major component of tannins possesses antimicrobial activity [34]. Furthermore, they showed that its antimicrobial mode of action was via dissolution of bacterial cell wall lipids which induces cell leakage and apoptosis [34].

In addition to the crude phytochemical screening, we also utilized GS-MS to evaluate bioactive compounds. The result showed more bioactive compounds via GC-MS compared to the crude extraction method. The compounds included trichloromethane, bicyclo[4.1.0]heptan-2-one 6-methyl, 1-tetradecyne, citronellylisobutyrate, hexadecanoic acid, methyl ester, 9-octadecenoic acid methyl ester (E)-, 1-methyl-4-phenyl-5-thioxo-1,2,4-triazolidin-3-one and carbonic acid 2-dimethylaminoethyl propyl ester among others. In an earlier study, 9-octadecenoic acid, methyl ester, and linoleic acid were shown to have various desirable properties namely anti-acne, antihistaminic, anti-eczemic properties in addition to acting as a hypo-cholesterolemic, 5-alpha reductase inhibitor [35]. In another study, 1,2-bis (trimethylsilyl) benzene is reportedly a plasticizer compound with antimicrobial and antifouling properties [36]. Venkata-Raman et al. [37] reported that hexadecanoic acid methyl ester, a fatty acid ester that possesses haemolytic 5-alpha reductase inhibitor potentials as well as other properties including lubricative, anti-androgenic, nematicidal, and pesticidal. Conversely, Gavamukulya et al. [31] reported twenty-five (25) bioactive compounds from the chromatogram of *A. muricata* extract while in our study *A. muricata* leaf revealed twenty-three (23) bioactive compounds including esters and aromatic-nitro compounds whose synergistic effect may be responsible for the antimicrobial potentials observed in extracts of *A. muricata* against *S. aureus*. In addition to their synergistic effect, studies indicates that the type of solvent drives the potency of the extract as well [38, 39]. This may be due to the ability of ethanol to have a favourable polarity index, thereby enhancing the extraction of the bioactive compounds in this plant. This observation is in line with previous reports on the potency of ethanol extract of *A. muricata* leaf [40–42]. Although, the molecular mechanisms of action of the leave extract of *A. muricata* are yet to be ascertained; however, its high antimicrobial activity against *S. aureus* observed in this study was consistent with earlier reports [43]. *S. aureus* strains employed in this study showed significant sensitivity to varying concentrations of the extract of *A. muricata* leaves that was slightly lower than that of cefoxitin used as control. This observation was in line with earlier reports and further confirms the potency of the extracts of *A. muricata* leaves in the management of *S. aureus*-related infections and diseases [43].

According to Gruszczyk et al. [20] capsular protein-5O (Cap5O) belongs to the UDP-glucose/GDP-mannose dehydrogenase family, a group of enzymes that catalyse the NAD-dependent 2-fold oxidation of alcohol (of UDP-N-acetyl-mannosamine) to an acid. Thus, Cap5O is reportedly important for the synthesis of serotype 5 CPS, responsible for preventing the interaction of bacteria with eukaryotic phagocytes and non-phagocytes. In the presence of Cap5O, bacterial interaction with eukaryotic cells is limited, and as such, host immune reactions including the polymorphonuclear leukocytes phagocytosis that induces the production of reactive oxygen species are brought to a halt [44]. In this study, the antimicrobial activity observed following treatment with the plant extracts showed a reduction in microbial load loosely suggesting that these phytochemicals could synergistically have antimicrobial activity as evident by the favourable binding poses recorded by some of the bioactive compounds. From the docking outputs, trichloromethane, bicyclo[4.1.0]heptan-2-one 6-methyl and 1-methyl-4-phenyl-5-thioxo-1,2,4-triazolidin-3-one both interacted with the N-terminal NAD-binding domain which comprises of amino acids residues numbered 1–150. By binding to this subunit of the protein, they could disrupt the essential energy metabolism role of this co-factor. On the other hand, carbonic acid monoamide N-(2-ethylphenyl) propyl ester interacted with both the N-terminal NAD-binding domain as well as C-terminal substrate binding domain (amino acids 197–301) via it amino acid residues which were glycine (GLY12), Isoleucine (ILL11), alanine (ALA 79), cysteine (258) and leucine (LEU 259). In their study, Gruszczyk et al. [20] showed that oxydo/reduction of the catalytic Cys-258 could control Cap5O activity and also proposed that regulation of the protein can be achieved using both tyrosine phosphorylation and reversible reduction of a disulfide bond involving the catalytic residue Cys-258. By binding with the catalytic residue CYS-258, carbonic acid monoamide N-(2-ethylphenyl) propyl has the potential to interfere with Cap5O catalytic activity.

Furthermore, the binding of the bioactive compounds to the Cap5O protein could potentially limit its ability to evade the human immune system, thus corroborating earlier reports that showed *A. muricata* leaves possesses antimicrobial potentials, anti-tumour, anti-inflmmatory, insecticidal, and nematicidial, properties among others [45, 46]. According to Lipinski’s rule of five (5), poor absorption or permeation for a predicted compound is most likely to be the case if certain conditions abound. These conditions include the new molecular entities having more than 5 hydrogen (H) bond donors, 10 H-bond acceptors, molecular weight greater than 500, and calculated Log P greater than 5 [47]. Interestingly, all four non-toxic bioactive compounds met Lipinski’s rule for the new molecular entities. This implies that these compounds will be better absorbed in-vivo.

The present study has a limitation. Our study was limited to only one CPS and we did not establish whether or not, the four bioactive compounds could be substrates for efflux transporters that abound in resistant pathogens. Future studies should be targeted at Cap5O and other CPS to completely understand how this pathogen evades the host immune system and design better drug candidates against *S. aureus.*

## Conclusion

The crude extract of *A. muricata* leaves possessed phytochemical and slightly higher than allowable limits of anti-nutrients in leafy vegetables. In line with previous studies, the extract reduced the microbial load of *S. aureus* isolates with zones that were comparable to those of Cefoxitin. The bioactive compounds of *A. muricata* leaves showed favourable interactions against the *S. aureus* CPS. By binding favourably to the Cap5O, it has the potential to interfere with the catalytic reaction that leads to the synthesis of CPS which drives the evasion of the human immune system. Synergistically or individually, these bioactive compounds could be a favorable drug target considering Cap5O importance in the production of CPS.

## Supplementary Information

**Additional file 1: Table S1.** Basic properties. **Table S2.** Advanced properties

## Data Availability

Data sets generated in this study are available from the corresponding author on request.

## Abbreviations

AA: Amino Acid
CFU: Colony forming unit
CLSI: Clinical Laboratory Standard Institute
CPS: Capsular polysaccharides
GC-MS: Gas chromatograph-mass spectrophotometer
NAD: Nicotinamide adenine dinucleotide (NAD)
NIST: National institute of standards and technology
MSA: Mannitol salt agar
PDB: Protein Data Bank
RCSB: Research collaboratory for structural bioinformatics
*S. aureus*: *Staphylococcus aureus*
mg: Milligram
mm: Millimeter
nm: Nanometer
rpm: Revolutions per minute
^o^C: Degree Celsius

## References

[1] Hiramatsu K, Katayama Y, Matsuo M, Sasaki T, Morimoto Y, Sekiguchi A, Baba T (2014). Multi-drug-resistant *Staphylococcus aureus* and future chemotherapy. J Infect Chemother.

[2] Harkins CP, Pichon B, Doumith M, Parkhill J, Westhhttps H, Tomasz A, de Lencastre H, Bentley SD, Kearns AM, Holden M (2017). Methicillin-resistant *Staphylococcus aureus* emerged long before the introduction of methicillin into clinical practice. Genome Biol.

[3] Mbim EN, Mboto CI, Edet UO (2016). Plasmid profile analysis and curing of multidrug resistant Bacteria isolated from two hospital environments in Calabar Metropolis, Nigeria. Asian J Med Health.

[4] Abubakar U, Sulaiman SAS (2018). Prevalence, trend and antimicrobial susceptibility of MethicillinResistant Staphylococcus aureus in Nigeria: a systematic review. J Infect Public Health.

[5] Kot B, Wierzchowska K, Piechota M, Grużewska A (2020). Antimicrobial resistance patterns in methicillin-resistant *Staphylococcus aureus* from patients hospitalized during 2015–2017 in hospitals in Poland. Med Princ Pract.

[6] Ahmadi E, Khojasteh M, Mortazavi S (2019). Prevalence of and risk factors for methicillin-resistant *Staphylococcus aureus* nasal carriage in the west of Iran: a population-based cross-sectional study. BMC Infect Dis.

[7] da Silva JB, Espinal M, Ramón-Pardo P (2020). Antimicrobial resistance: time for action. Rev Panam Salud Publica.

[8] World Health Organization. WHO publishes list of Bacteria for which new antibiotics are urgently needed. Available at: https://www.who.int/news/item/27-02-2017-who-publishes-list-of-bacteria-for-which-new-antibiotics-are-urgently-needed Accessed 01 Feb 2022.

[9] Ebana RUB, Andy IE, Edet UO, Benjamin AU, Mbim EN, Anosike IK (2019). Nutritional studies and antimicrobial activities of *Jatropha tanjorensis* leaves extracts against *Escherichia coli* isolates. Int J Innov Sci Res Technol.

[10] Ebana RUB, Asamudo NU, Etok CA, Edet UO, Onyebuisi CS (2016). Phytochemical screening, nutrient analysis and antimicrobial activity of the leaves of *Lasianthera africana* and *Dennettia tripetala* on clinical isolates. J Adv Biol Biotechnol.

[11] Edet UO, Mboto CI, George UE, Umego CF (2017). Comparative evaluation of the effect of *Annona muricata* (Graviola) leaves extracts and Cefoxitin on *Staphylococcus aureus*. Asian J Biol.

[12] Anand U, Jacobo-Herrera N, Altemimi A, Lakhssassi N (2019). A comprehensive review on medicinal plants as antimicrobial therapeutics: potential avenues of biocompatible drug discovery. Metabolites..

[13] Anand U, Nandy S, Mundhra A, Das N, Pandey DK, Dey A (2020). A review on antimicrobial botanicals, phytochemicals and natural resistance modifying agents from Apocynaceae Family: possible therapeutic approaches against multidrug resistance in pathogenic microorganisms. Drug Resist Updat.

[14] Khare T, Anand U, Dey A, Assaraf YG, Chen ZS, Liu Z, Kumar V (2021). Exploring phytochemicals for combating antibiotic resistance in microbial pathogens. Front Pharmacol.

[15] Mbaveng AT, Chi GF, Bonsou IN, Ombito JO, Yeboah SO, Kuete V, Efferth T (2021). Cytotoxic phytochemicals from the crude extract of Tetrapleura tetraptera fruits towards multi-factorial drug resistant cancer cells. J Ethnopharmacol.

[16] Mohammed HA, Al-Omar MS, Mohammed SAA, Alhowail AH, Eldeeb HM, Sajid MSM, Abd-Elmoniem EM, Alghulayqeh OA, Kandil YI, Khan RA (2021). Phytochemical analysis, pharmacological and safety evaluations of halophytic plant, *Salsola cyclophylla*. Molecules..

[17] Neglo D, Tettey CO, Essuman EK, Amenu JD, Mills-Robertson FC, Sedohia D, et al. Evaluation of the modulatory effect of *Annona muricata* extracts on the activity of some selected antibiotics against biofilm-forming MRSA. Evid Based Complement Alternat Med. 2021:9342110. 10.1155/2021/9342110. 10.1155/2021/9342110 PMC8712150 34966438

[18] Pinzi L, Rastelli G (2019). Molecular docking: shifting paradigms in drug discovery. Int J Mol Sci.

[19] Torres PHM, Sodero ACR, Jofily P, Silva-Jr F (2019). Key topics in molecular docking for drug design. Int J Mol Sci.

[20] Gruszcyk J, Fleurie A, Olivares-IIIana V, Bechet E, Zanella-Cleon I, Morera S, Meyer P, Pompidor G, Kahn R, Grangeasse C (2011). Structure analysis of the *Staphylococcus aureus* UDP-*N*acetyl- mannosamine dehydrogenase Cap5O involved in capsular polysaccharide biosynthesis. J Biol Chem.

[21] Chan YG-Y, Kim HK, Schneewind O, Missiakas D (2014). The capsular polysaccharide of *Staphylococcus aureus* is attached to peptidoglycan by the LytR-CpsA-Psr (LCP) family of enzymes. J Biol Chem.

[22] Rausch M, Deisinger JP, Ulm H, Muller A, Li W, Hardt P, Wang X, Li X, Sylvester M, Engeser M, Vollmer W, Muller CE, Sahl HG, Lee JC, Schneider T (2019). Coordination of capsule assembly and cell wall biosynthesis in *Staphylococcus aureus*. Nat Commun.

[23] Trease GE, Evans WC (1982). A textbook of Pharmacolognosy 11^th^ edition Bailliese, Tindall, London.

[24] Sofowara EA (1982). Medicinal plants and tradition medicine in Africa.

[25] Uma KS, Parthiban P, Kalpana S (2017). Pharmacognostical and preliminary phytochemical screening and extraction of Aavaarai Vidhai Chooranam. Asian J Pharm Clin Res.

[26] Tiwari P, Kumar B, Kaur M, Kaur G, Kaur H (2011). Phytochemical screening and extraction. Int Pharm Sci.

[27] Auwal MS, Saka S, Mairiga IA, Sanda KA, Shuaibu A, Ibrahim A (2014). Preliminary phytochemical and elemental analysis of aqueous and fractionated pod extracts of *Acacia nilotica* (Thorn mimosa). Vet Res Forum.

[28] Njoku OV, Obi C (2009). Phytochemical constituents of some selected medicinal plants. Afr J Pure Appl Chem.

[29] Dye WB (1956). Studies on *Halogetonglomeratus*. Weeds.

[30] Clinical Laboratory Standard Institute (2012). Performance standards for antimicrobial susceptibility testing; Twenty-second Informational Supplement.

[31] Gavamukulya Y, Abou-Elella F, Wamunyokoli F, El-Shemy H (2015). GC-MS analysis of bioactive phytochemicals present in Ethanolic extracts of leaves of *Annona muricata*: a further evidence for its medicinal diversity. Pharmacognosy J.

[32] Pai BHM, Rajesh G, Shenoy R, Rao A (2016). Anti-microbial efficacy of Soursop leaf extract (*Annona muricata*) on Oral pathogens: an in-vitro study. J Clin Diagn Res.

[33] Bhagavathy S, Mahendiran C, Kanchana R (2019). Identification of glucosyl transferase inhibitors from Psidium guajava against Streptococcus mutans in dental caries. J Tradit Complement Med.

[34] Daglia M (2011). Phytochemical and elemental analysis of *Acalypha wilkesiana* leaf. J Am Sci.

[35] Ganesh M, Mohankumar M (2017). Extraction and identification of bioactive components in *Sida cordata* (Burm.F.) using gas chromatography-mass spectrometry. J Food Sci Technol.

[36] Thomas E, Thomas DG, Anandan R (2013). GC-MS analysis of phytochemical compounds present in the rhizomes of Nerviliaaragoana GAUD. Asian J Pharm Clin Res.

[37] Venkata-Raman B, Samuel LA, Pardha SM, Narashimha RB, Naga VKA, Sudha-kar M, Badhakrishnan TM (2012). Antibacterial, antioxidant activity and GC-MS analysis of Eupatorium odoratum. Asian J Pharm Clin Res.

[38] Coria-Téllez AV, Montalvo-Gónzalez E, Yahia EM, Obledo-Vázquez EN (2018). *Annona muricata*: a comprehensive review on its traditional medicinal uses, phytochemicals, pharmacological activities, mechanisms of action and toxicity. Arab J Chem.

[39] Abdul Wahab SM, Ibrahim J, Areeful HM, Laiba A. Exploring the leaves of Annona muricata L. as a source of potential anti-inflammatory and anticancer agents. Front Pharmacol. 2018;9 https://www.frontiersin.org/article/10.3389/fphar.2018.00661.DOI=10.3389/fphar.2018.00661. 10.3389/fphar.2018.00661 PMC6019487 29973884

[40] Ganesh M, Mohankumar M (2017). Extraction and identification of bioactive components in *Sida cordata* (Burm.F.) using gas chromatography-mass spectrometry. J Food Sci Technol.

[41] Vijayameena C, Subhashini G, Loganayagi M, Ramesh B (2013). Phytochemical screening and assessment of antibacterial activity for the bioactive compounds in *Annona muricata*. Int J Curr Microbiol App Sci.

[42] Moghadamtousi SZ, Fadaeinasab M, Nikzad S, Mohan G, Ali M, Kadir AH (2015). *Annona muricata* (Annonaceae): a review of its traditional uses, isolated acetogenins and biological activities. Int J Mol Sci.

[43] Abdulsalami MS, Aina VO, Ibrahim MB, Adejo GO, Audu G (2016). ComparativeAntibacterial study of aqueous and Ethanolic leaf extracts of *Annona muricata*. J Nat Sci Res.

[44] Voyich JM, Braughton KR, Sturdevant DE, Whitney AR, Saïd-Salim B, Porcella SF, Long RD, Dorward DW, Gardner DJ, Kreiswirth BN, Musser JM, DeLeo FR (2005). Insights into mechanisms used by *Staphylococcus aureus* to avoid destruction by human neutrophils. J Immunol.

[45] Pinto NDC, Campos LM, Evangelista ACS, Lemos ASO, Silva TP, Melo RCN, de Lourenco CC, Salvador MJ, Apolonio ACM, Scio E, Fabri R (2017). Antimicrobial *Annona muricata* L. (soursop) extract targets the cell membranes of gram-positive and gram-negative bacteria. Ind Crop Prod.

[46] Benet LZ, Hosey CM, Ursu O, Oprea TI (2016). BDDCS, the rule of 5 and drugability. Adv Drug Deliv Rev.

[47] Lawal ZA, Hamid AA, Shehu A, God’shelp E, Ajibade OS, Zubair OA, Ogheneovo P, Mukadam AA, Adebayo CT (2017). Biochemical properties, in-vitro antimicrobial, and free radical scavenging activities of the leaves of Annona muricata. J Appl Sci Environ Manag.

