# Supplementary material for: Evaluation of Annona muricata extract against Staphylococcus aureus isolate and in-silico activity of bioactive compounds against Capsular protein (Cap5O)
Source: BMC Complement Med Ther. 2022 Jul 19;22:192. doi: 10.1186/s12906-022-03672-4 (PMC9297590; doi:10.1186/s12906-022-03672-4)
Supplement: Supplementary file 1 — Additional file 1: Table S1. Basic properties. Table S2. Advanced properties [file 12906_2022_3672_MOESM1_ESM.docx]

**Table S1: Basic properties**

| **Basic property** | Trichloromethane | Bicyclo[4.1.0]heptan-2-one, 6-methyl- | 1-methyl-4-phenyl-5-thioxo-1,2,4-triazolidin-3-one | - Carbonic acid, monoamide, N-(2-ethylphenyl)-, propyl ester |
| --- | --- | --- | --- | --- |
|  | **Values** | | | |
| Mass | 119.3731 | 124.1799 | 207.2546 | 175.2254 |
| logP | 1.9864 | 1.7656 | 1.2336 | 1.1112 |
| H-bond acceptors | 0 | 1 | 4 | 4 |
| H-bond donors | 0 | 0 | 1 | 0 |
| Rotatable bonds | 0 | 0 | 1 | 7 |
| PSA | 0 | 17.0700 | 74.8100 | 38.7700 |
| RO5 violations | 0 | 0 | 0 | 0 |
| RO3 violations | 0 | 0 | 2 | 2 |
| Refractivity | 21.3090 | 36.2820 | 56.4757 | 46.2250 |
| Atoms | 5 | 21 | 23 | 29 |
| Rings | 0 | 2 | 2 | 0 |
| Heavy atoms | 4 | 9 | 14 | 12 |
| Hydrogen atoms | 1 | 12 | 9 | 17 |
| Heteroatoms | 3 | 1 | 5 | 4 |
| N/O atoms | 0 | 1 | 4 | 4 |
| Inorganic atoms | 0 | 0 | 0 | 0 |
| Halogen atoms | 3 | 0 | 0 | 0 |
| Chiral centers | 0 | 2 | 0 | 0 |
| R/S chiral centers | 0 | 0 | 0 | 0 |
| Unknown chiral centers | 0 | 0 | 0 | 0 |
| Undefined chiral centers | 0 | 2 | 0 | 0 |
| Stereo double bonds | 0 | 0 | 0 | 0 |
| Cis/trans stereo double bonds | 0 | 0 | 0 | 0 |
| Unknown stereo double bonds | 0 | 0 | 0 | 0 |
| Undefined stereo double bonds | 0 | 0 | 0 | 0 |

**Table S2: Advanced properties**

| **Advanced**  **property** | Trichloromethane | Bicyclo[4.1.0]heptan-2-one, 6-methyl- | 1-methyl-4-phenyl-5-thioxo-1,2,4-triazolidin-3-one | - Carbonic acid, monoamide, N-(2-ethylphenyl)-, propyl ester |
| --- | --- | --- | --- | --- |
|  | **Values** | | | |
| logP | 1.8300 | 1.7100 | 1.7300 | 1.4400 |
| logD (at pH=7.4) | 1.8300 | 1.7100 | 1.7300 | 0.3900 |
| Negative charges (at pH=7.4) | 0 | 0 | 0 | 0 |
| Positive charges (at pH=7.4) | 0 | 0 | 0 | 1 |
| Total charge (at pH=7.4) | 0 | 0 | 0 | 1 |
| Number of charges (at pH=7.4) | 0 | 0 | 0 | 1 |
| Strongest acidic pKa |  |  | 13.6500 |  |
| Strongest basic pKa |  | -7.4200 | -9.2500 | 8.4100 |
| Isoelectric point |  |  |  |  |
| Mass | 119.3700 | 124.1830 | 207.2500 | 175.2280 |
| ASA (at pH=7.4) | 240.2900 | 280.0200 | 351.3900 | 483.7300 |
| ASA+ (at pH=7.4) | 54.9400 | 192.9100 | 214.4500 | 341.2200 |
| ASA- (at pH=7.4) | 185.3600 | 87.1200 | 136.9400 | 142.5100 |
| ASA_H (at pH=7.4) | 219.9200 | 252.1200 | 306.2500 | 415.3500 |
| ASA_P (at pH=7.4) | 20.3700 | 27.9000 | 45.1400 | 68.3800 |
| Bonds | 4 | 22 | 24 | 28 |
| Chiral centers | 0 | 2 | 0 | 0 |
| PSA (at pH=7.4) | 0 | 17.0700 | 35.5800 | 39.9700 |
| Rotatable bonds | 0 | 0 | 1 | 7 |
| H-bond acceptors (at pH=7.4) | 0 | 1 | 1 | 2 |
| H-bond donors (at pH=7.4) | 0 | 0 | 1 | 1 |
| RO5 violations (at pH=7.4) | 0 | 0 | 0 | 0 |
| RO3 violations (at pH=7.4) | 0 | 0 | 0 | 1 |
